# Rejuvenation of Neutrophil Functions in Association With Reduced Diabetes Risk Following Ten Weeks of Low-Volume High Intensity Interval Walking in Older Adults With Prediabetes – A Pilot Study

**DOI:** 10.3389/fimmu.2020.00729

**Published:** 2020-05-05

**Authors:** David B. Bartlett, Cris A. Slentz, Leslie H. Willis, Andrew Hoselton, Janet L. Huebner, Virginia B. Kraus, Jennifer Moss, Michael J. Muehlbauer, Guillaume Spielmann, Deborah M. Muoio, Timothy R. Koves, Helena Wu, Kim M. Huffman, Janet M. Lord, William E. Kraus

**Affiliations:** ^1^Duke Molecular Physiology Institute, School of Medicine, Duke University, Durham, NC, United States; ^2^Division of Medical Oncology, School of Medicine, Duke University, Durham, NC, United States; ^3^MRC-ARUK Centre for Musculoskeletal Ageing Research, Institute of Inflammation and Ageing, University of Birmingham, Birmingham, United Kingdom; ^4^Department of Kinesiology, Louisiana State University, Baton Rouge, LA, United States; ^5^NIHR Birmingham BRC in Inflammation, University Hospitals Birmingham, Birmingham, United Kingdom

**Keywords:** glucose control, high intensity interval training, immune function, immunometabolism, neutrophils, prediabetes

## Abstract

Neutrophil dysfunction is a common feature of aging, and is associated with the pathogenesis of many age-related diseases, including type 2 diabetes mellitus (T2DM). Although exercise training improves metabolic health, decreases risk of T2DM, and is associated with improving neutrophil functions, involvement in regular physical activity declines with age. The aim of this study was to determine if neutrophil functions could be improved in association with changes in fitness and metabolic parameters in older adults at risk for T2DM using 10-weeks of low volume high-intensity interval exercise training (HIIT). Ten older (71 ± 5 years) sedentary adults with prediabetes (HbA1c: 6.1 ± 0.3%) completed 10 weeks of a supervised HIIT program. Three 30 min sessions/week consisted of ten 60 s intervals of low intensity [50–60% heart rate reserve (HRR)] separated with similar durations of high intensity intervals (80–90% HRR). Before and after training, glucose and insulin sensitivity, neutrophil chemotaxis, bacterial phagocytosis, reactive oxygen species (ROS) production, and mitochondrial functions were assessed. Exercise-mediated changes in cardiorespiratory fitness (VO_2__peak_) and neutrophil functions were compared to six young (23 ± 1 years) healthy adults. Following training, significant reductions in fasting glucose and insulin were accompanied by improved glucose control and insulin sensitivity (all *p* < 0.05). Before exercise training, VO_2__peak_ in the old participants was significantly less than that of the young controls (*p* < 0.001), but increased by 16 ± 11% following training (*p* = 0.002) resulting in a 6% improvement of the deficit. Neutrophil chemotaxis, phagocytosis and stimulated ROS production were significantly less than that of the young controls, while basal ROS were higher before training (all *p* < 0.05). Following training, chemotaxis, phagocytosis and stimulated ROS increased while basal ROS decreased, similar to levels observed in the young controls (all *p* < 0.05) and reducing the deficit of the young controls between 2 and 154%. In five of the adults with prediabetes, neutrophil mitochondrial functions were significantly poorer than the six young controls before training. Following training, mitochondrial functions improved toward those observed in young controls (all *p* < 0.05), reducing the deficit of the young controls between 14.3 and 451%. Ten weeks of HIIT in older adults at risk for T2DM reduced disease risk accompanied by improved primary and bioenergetic neutrophil functions. Our results are consistent with a reduced risk of infections mediated by relationships in exercise induced systemic and cellular metabolic features.

**Clinical Trial Registration:** www.ClinicalTrials.gov, identifier NCT02441205, registered on May 12th, 2015.

## Introduction

Neutrophil dysfunction is a common feature of aging and is associated with the pathogenesis of age-related diseases, including type 2 diabetes mellitus (T2DM) (1, 2). In type 2 diabetes, almost all functions of neutrophils are impaired, including phagocytosis, pathogen killing, and chemotaxis (3). Individuals with type 2 diabetes, and those at risk of type 2 diabetes, have an increased risk for bacterial infections such as *Escherichia coli*, *salmonella*, and *helicobacter* (4). Additionally, basal production of pro-inflammatory cytokines and reactive oxygen species (ROS) are elevated, contributing to increased pathogenic chronic low-grade systemic inflammation (5). Chronic inflammation and neutrophil dysfunction are implicitly linked. Causal to this are the metabolic perturbations of type 2 diabetes, including increased free fatty acids, insulin resistance and hyperglycemia. These metabolic perturbations are associated with altered neutrophil glycolysis, glutaminolysis, lipid oxidation, and mitochondrial functions, leading to impaired primary functions (3).

Exercise training improves metabolic health, reduces inflammation, and decreases the risk of type 2 diabetes in adults with prediabetes (6, 7). Although some of the benefits can be attributed to reductions in adipose tissue, exercise has beneficial effects on immune cell phenotype and functions (8). In our work, greater levels of physical activity in healthy older adults are associated with better neutrophil chemotaxis (9). Further, as compared to moderate intensity continuous training, neutrophil bactericidal functions are comparably improved in sedentary adults following 10 weeks of low-volume high-intensity interval training (HIIT) (10). HIIT may be a useful strategy for reducing disease risk in older adults due to its time-efficient approach and high adherence in older adults (11, 12). Robinson and colleagues suggest that in adults (52 ± 10 years) with prediabetes, only 10 days of HIIT improves cardiorespiratory fitness and alters phenotypic properties of monocytes (13). Although HIIT did not reduce fasting glucose, the comparison group of moderate-intensity continuous training (MICT) did observe a reduction in glucose (13). Others have shown that short periods of HIIT can improve indices of glycemic control (14–16). In obese (BMI: 37.8 ± 1.3 kg/m^2^) women (48 ± 2 years), 2 weeks of low-calorie dieting with 12 sessions of 60 min/day interval training increased insulin sensitivity (14). Although body fat was reduced, cardiorespiratory fitness did not increase, suggesting a role for adipose induced glycemic changes independent of cardiorespiratory fitness. In another study, Gilbertson and colleagues suggested that prediabetes was reversed in 40% of a slightly older (60.9 ± 1.4 years), lower BMI (33.5 ± 1.1 kg/m^2^), small (*n* = 31) cohort of men (*n* = 7) and women with prediabetes, following 12 sessions of exercise (16). Exercise sessions were either 60 min of HIIT or continuous exercise, matched on workload as defined by average percentage of peak heart rate. Although exercise did not reduce fasting glucose, total area under the curve for glucose OGTT was reduced in both groups, with minimal changes in body composition. Heiskanen and colleagues suggested that 2 weeks of supramaximal sprint intervals (SIT) or MICT can reduce body fat content, improve beta cell function, and lower HbA1c in men and women (40–55 years) with prediabetes or type 2 diabetes (15). In this study, fasting glucose did not change with exercise, and HbA1c changes were a likely consequence of hemodilution due to increased plasma volume (17). As such, whether short-term exercise training truly improves glucose homeostasis remains unknown. In the morbidly obese, as in the Gilbertson and colleagues study population, perhaps it does, but dietary caloric restriction appears to be driving this effect (14). Further, few studies have assessed the effects of longer exposure (>6 weeks) to HIIT or SIT in truly older (>65 years) populations, whether with prediabetes or not (12). Therefore, to test whether HIIT could improve glucose control and insulin sensitivity in older adults with prediabetes we opted to utilize and modify our previous 10 week HIIT program (10, 11, 18). Ten weeks of HIIT, prescribed using percentages of VO_2__peak_, was sufficient to improve glucose homeostasis, cardiorespiratory fitness, and innate immune functions in younger, middle-aged, and older adults (10, 11, 18). Importantly, our program is designed to have minimal effects on body composition (i.e., lean mass and fat mass), which would potentially confound our results.

The aim of this exploratory study was to examine the effects of 10 weeks of HIIT on cardiorespiratory fitness, glucose control, systemic inflammation, and neutrophil functions in older adults with prediabetes. We utilized a HIIT program that was walking in nature, and elicits minimal changes in body composition to ensure that effects would be mostly associated with changes in fitness. Because cardiorespiratory fitness reduces approximately 10% per decade of life, any increases in fitness will reflect a rejuvenation (i.e., old to younger) of cardiorespiratory fitness. Therefore, to determine the degree of potential HIIT-mediated immune cell rejuvenation, we chose to compare results with a young healthy control group – in effect a positive control. We hypothesized that in older adults with prediabetes, 10 weeks of three sessions/week of HIIT walking would improve cardiorespiratory fitness, neutrophil primary functions and bioenergetics, and reduce diabetes risk factors.

## Materials and Methods

### Participants

Ten old (71 ± 5 years) sedentary participants (four men and six women) with prediabetes (HbA1c of 6.1 ± 0.3%) were recruited for the study. Participants were free of cancer, cardiovascular disease, or autoimmune disease, and were physically inactive. All women were post-menopausal and were not receiving hormone replacement therapy. All participants were medication stable. As a control group to assess exercise-mediated associations with age-related declines in immune function and aerobic fitness, six healthy young adults (three men and three women; 23 ± 1 years) were recruited. Young healthy controls were recruited from individuals who worked at the Duke Center for Living, were free from cardiometabolic or autoimmune disease, and were all considered highly active, engaging in >3 days/week of structured exercise training. All measured indices of the controls were deemed normal and healthy: BMI (25 ± 2.6 kg/m^2^), body fat (18 ± 10.8%), SBP (112 ± 4 mmHg), DBP (70 ± 4 mmHg), fasting glucose (4.2 ± 0.3 mmol/L), fasting insulin (68.1 ± 26.2 pmol/L), and white blood cell (4.9 ± 0.4 × 10^9^/L) and neutrophil counts (3.1 ± 0.2 × 10^9^/L). All participants gave written informed consent and the study was approved by the Duke University Medical Center Institutional Review Board (IRB#: Pro00055208 and Pro00078608).

### Fitness, Function and Body Composition

Exercise treadmill testing was used to assess cardiorespiratory fitness. Aerobic capacity (VO_2__peak_) was determined by a graded maximal treadmill test starting at 2 mph/0% grade and then increasing speed and/or grade such that the metabolic demand increased at approximately 3.5 mL/kg/min until volitional exhaustion. A valid test was confirmed by either an RER of >1.1 (mean RER: 1.2 ± 0.1 at both times) or a rating of perceived exertion of ≥17 (mean: 18 ± 1). Body weight, fat mass and lean mass were determined by air-displacement plethysmography (BodPod System; Life Measurement Corporation, Concord, CA, United States). Resting blood pressure and heart rate were taken following 15 min of sitting. Each participant also completed a short battery of physical functioning tests which are indicative of health and frailty in older adults (19, 20). Grip strength was assessed by dynamometry in both hands in triplicate; the best score was taken. Timed-up and go, Berg balance scale, and 400 m walk tests were all conducted by a trained exercise physiologist. All tests were completed before training and at least 24 h after the last exercise session.

### Exercise Training

Exercise training consisted of 10 weeks of 3 × 30-min sessions per week of supervised treadmill walking. In aggregate, participants completed >90% of prescribed exercise sessions, Table 1. Exercise intensities were determined from a cardiorespiratory fitness test. For exercise prescription, VO_2_ reserve was used and calculated as previously described (21). Participants were given between 3 and 6 sessions to become accustomed to the exercise (30- to 45-s intervals at target heart rates, total time 20 min). Exercise consisted of a 5-min warm-up and 5-mine cool down as part of the total session. Intervals were designed to elicit a heart rate corresponding to 80–90% of VO_2_ reserve (high intensity intervals) and 50–60% VO_2_ reserve (active recovery). Speeds did not exceed walking pace (range: 1–4.6 mph) and if heart rate was not achieved by walking speed, gradient (range 1–15%) was added to increase heart rate. High intensity intervals were between 60 and 90 s followed by active recovery intervals of a similar duration. Rather than controlling each session for energy expenditure, total intervals per session were adjusted so that the exercise duration per session was 30 min. Ratings of perceived exertion were detailed at the end of each high intensity interval bout.

**TABLE 1:** Exercise prescription.

	**High-intensity interval**	**Low-intensity interval**
**Prescribed**		
Intensity (% VO_2peak_)	80–90	50–60
Heart rate (bpm)	133 ± 15 – 141 ± 16	108 ± 11 – 116 ± 12
**Duration**		
Each interval (sec)	60	60
Warm-up + Cool down (min)	10	
Total Ex. Time/Week (min)	90	
Total sessions (*N)*	30	
Total exercise exposure (min)	300	300
**Actual**		
Intensity (% VO_2peak_)	87 ± 6	70 ± 5
Estimated EE (Kcals/week)	401.5 ± 108.8	
Heart rate (bpm)	139 ± 18	125 ± 15
**Duration**		
Each interval (%Rx)	97 ± 3	100
Total Ex. Time/week (%Rx)	98 ± 2	
Total sessions (%Rx)	97 ± 3	

*EE, energy expenditure.*

### Complete Blood Differentials

Complete blood differential counts were measured in EDTA treated whole blood using a hematology analyzer (Sysmex, United States).

### Immune Cell Isolation

Neutrophils were isolated within 1 h of collection, from heparin-treated blood following 2% dextran sedimentation and separation on a discontinuous Percoll gradient as previously described (9). Neutrophil purity and viability were determined by Giemsa staining (Diff-Quik; Gentaur Europe, Brussels, Belgium) and trypan blue exclusion, respectively. Neutrophil purity and viability were consistently ≥95%. Depending on the assay, neutrophils were re-suspended at 3 × 10^6^ cells/mL in either RPMI 1640 medium only (Sigma-Aldrich, United States), RPMI 1640 containing 0.15% bovine serum albumin (BSA; Sigma-Aldrich) or HBSS+/+ (+calcium, +magnesium) medium.

### Neutrophil Migration

Neutrophil migratory dynamics were assessed using an Insall chamber (Weber Scientific International Ltd., Teddington, United Kingdom) as previously described (22, 23). Briefly, coverslips were coated with 7.5% culture tested BSA (Sigma-Aldrich, St. Louis, MO, United States) and neutrophils adhered to this surface for 20 min at room temperature. The use of this BSA has previously been shown to mimic the ligand for CD11b and CD18, ICAM-1 (24). The coverslip was then inverted on the Insall chamber before addition of buffer (RPMI-1640) alone as a control or buffer containing 100 nM IL-8 (R&D Systems, Minneapolis, MN, United States). Neutrophil migration was monitored using a Zeiss Axiovert 100 inverted microscope (Zeiss, White Plains, NY, United States) fitted with a Hamamatsu ORCA 100 digital camera (Hamamatsu, Japan). Time-lapse recordings and calculation of neutrophil migratory dynamics were performed as previously described (22). Briefly, the Insall chamber allows the formation of stable chemoattractant gradients, with defined, consistent direction in the y direction for each experiment. Only distance traveled in the y direction over time was included in calculations of chemotaxis. Migration was assessed using three parameters: Average cell speed (μm/min) of movement toward the chemokine (termed chemokinesis), average velocity (μm/min) of cells (termed chemotaxis) and accuracy of movement (termed chemotactic index). Chemotactic index is expressed in a comparative scale and arbitrary unit (a.u.) ranging from −1 to +1. Movement directly toward the chemoattractant is +1 whilst directly away is −1. Recordings lasted 20 min per experiment, with 20 slides captured using Improvision OpenLab software. The Java software ImageJ (Wayne Rasband, NIH, Bethesda, MD, United States) was used to analyze cell tracks. All analyses were carried out by a single analyst, blinded to subject group and cell conditions.

### Bacterial Phagocytosis

Phagocytosis of opsonized FITC-labeled *E. coli* (ThermoFisher Scientific) was assessed in whole blood as previously described (9). Briefly, phagocytosis was assessed in heparin treated whole blood and incubated at 4°C or 37°C (test) with *E. coli*. Phagocytosis was halted by the addition of cold phosphate buffered saline (PBS) whilst cell surface bound FITC was quenched by addition of 1% trypan blue solution. Unbound free bacteria were removed by washing in PBS and erythrocytes lysed and leukocytes fixed using 1% fix/lyse solution (ThermoFisher Scientific). Cell DNA was counterstained by addition of propidium iodide (PI) before flow cytometry analysis was performed.

### Neutrophil ROS Production

Reactive oxygen species generation was assessed by luminol-amplified chemiluminescence as previously described (25). Briefly, resting neutrophils [1 × 10^5^ in HBSS+/+ (+calcium, +magnesium)] were dispensed into a 96-well white-bottom flat plate (Corning, United States) containing 1 μM luminol (pH 7.3; Sigma-Aldrich). Cells were stimulated with 25 nM PMA (test) or HBSS+/+ and immediately assessed for ROS generation at 1-min intervals for 60 min using a Tecan Infinite 200 Pro plate reader (Tecan, Mannedorf, Switzerland). Experiments were performed in triplicate, with ROS production measured as relative light units (RLU) and calculated as area under the curve (AUC).

### Neutrophil Elastase Activity

Plasma neutrophil elastase activity was assessed by fluorometric quantification using the manufacturer’s guidelines (Sigma). Briefly, in duplicate, 10 μL of plasma or standards are added to 40 μL of assay buffer before addition of 100 μL of substrate buffer. Elastase proteolytically cleaves the substrate to release a fluorophore that can be detected at an excitation wavelength of 380 nm and emission wavelength of 500 nm. Results are compared against a standard curve and concentration of neutrophil elastase calculated.

### Neutrophil Mitochondrial Function

As exploratory analyses, we assessed neutrophil mitochondrial function in 5 (three women and two men) of the HIIT participants before and after HIIT, and compared results to the young control adults. Neutrophil mitochondrial bioenergetics were determined using the Seahorse XF24 extracellular flux analyzer (Agilent Technologies), which measures oxygen flux and pH changes in media above a cell monolayer (26). Prior to assessing participant samples, pilot work determined the optimal number of neutrophils to be seeded in order to accurately measure oxygen consumption rate (OCR). There was a cell number dependent OCR response which plateaued at 1 × 10^6^ neutrophils, after which OCR responses became non-linear. Neutrophils were seeded in triplicate in 100 μL of XF assay media in wells containing Cell-Tak (BD Bioscience) for 35 min in a NON-CO_2_ incubator at 37°C. Plates were briefly spun and the monolayer checked by microscopy. Careful addition of 500 μL of XF assay media was completed and cells incubated for a further 40 min in a Non-CO_2_ incubator at 37°C. The following compounds were added to the appropriate ports on the sensor cartridge for a final concentration of: (A) oligomycin (1.5 μM), (B) Carbonyl cyanide-4-(trifluoromethoxy) phenylhydrazone [FCCP (0.5 μM)], (C) Rot/AA (0.5 μM). Following calibration, the cell culture plate was added to the Seahorse analyzer for a 3-h assay. Measurements were acquired every 15 min, with three measurements completed before injection of each compound. Following completion of each plate, cells were lysed and protein content assessed by the BCA method (ThermoFisher Scientific). Each well/plate was then normalized to total well protein content using standardized methods. Data were analyzed using Wave Desktop 2.6 software (Agilent).

Mitochondrial membrane potential (ΔΨm) of isolated neutrophils was assessed by JC-1 fluorescence using standardized methods (Cayman Chemicals, Ann Arbor, MI, United States). Briefly, 5 × 10^5^ isolated neutrophils were incubated at 37°C for 20 min in HBSS containing 2.5 μM of the lipophilic cationic dye, 5,5′,6,6′-tetrachlor-1,1′3,3′-tetraethylbenzimi-dazolylcarbocyanine iodide (JC-1). Cells were washed and plated in a clear bottom black 96-well plate. Fluorescence was measured in triplicate first at an excitation/emission of 535 nm/595 nm (aggregates) followed by 485 nm/535 nm (monomers) on an Infinite 200 PRO plate reader (Tecan Life Sciences, Männedorf, Switzerland). The JC-1 forms aggregates in healthy cells while apoptotic unhealthy cells suffer loss of ΔΨm rendering JC-1 in monomeric form. The ratio of aggregates: monomers was calculated and used as an index of ΔΨm. As a control, 5 μM of FCCP was used to disrupt ΔΨm which produces an increase in monomers and lowers the ratio of aggregates to monomers.

### Measurement of Neutrophil Surface Receptor Expression

Neutrophil surface receptor expression was assessed in whole blood. Briefly, 100 μL of heparin treated blood was dispensed into 5 mL tubes and stored at 4°C in the dark. Cells were stained with anti-CXCR2-PE (ThermoFisher, clone 5E8-C7-F10), anti-CD16-FITC (BD Bioscience, clone 3G8), anti-CD11b-APC (BD Bioscience, clone ICRF44), anti-CD18-PE (BD Bioscience, clone 6.7), anti-TLR2-Alexa-647 (BD Bioscience, clone 11G7) or anti-TLR4-APC (ThermoFisher, clone HTA-125) or their relevant concentration-matched isotype control for 30 min on ice in the dark. Following incubation, cells were washed twice in cold PBS and erythrocytes lysed and leukocytes fixed using 1% fix/lyse solution (ThermoFisher Scientific). Following fixation, cells were washed twice and resuspended in 300 μL PBS/1%BSA for analysis by flow cytometry. All flow cytometry analyses were conducted on a BD FACSCanto II (BD Bioscience, United States) flow cytometer equipped with 3-lasers using the Duke Cancer Institute Core Facility, which maintained daily quality controls of the machine. Analyses were completed on 10,000 neutrophils and data analyzed using FCS Express 6 (FCS Express, United States).

### Plasma Analyses

Samples were processed immediately for plasma isolation and samples stored at −80°C until analyzed. All plasma analyses were completed by the Core Facilities within the Duke Molecular Physiology Institute. Plasma samples obtained following an overnight fast were analyzed for 5 cytokines and 1 acute phase protein: IL-8, IL-6, IL-10, TNFα, IL-1β, and C-reactive protein (CRP). Concentrations of cytokines were determined in duplicate using a human pro-inflammatory 5-plex sandwich immunoassay according to the manufacturer’s instructions (Meso Scale Discovery, Rockville, MD, United States). The lower limits of detection in pg/mL were: IL-1β (0.03 pg/mL), IL-6 (0.11 pg/mL), IL-7 (0.15 pg/mL), IL-8 (0.08 pg/mL), IL-10 (0.05 pg/mL), IL-15 (0.12 pg/mL), TNFα (0.09 pg/mL), and CRP (0.02 mg/L). All samples had concentrations greater than the LLOD with the exception of IL-1β with 77% of samples above the LLOD. Plasma concentrations of non-esterified fatty acid (NEFA) were assessed by an enzymatic colorimetric assay on a Beckman UniCel DxC600 Analyzer using the manufacturer’s guidelines (Wako Diagnostics, Richmond, VA, United States). High sensitivity CRP was measured in duplicate using a commercially available ELISA (IBL International, Germany). Leptin and adiponectin were assessed in duplicate using a commercially available ELISA (R&D Systems, Minneapolis, MN, United States). Adiponectin measured consisted of all three isoforms (low, middle, and high molecular weight).

### Glucose and Insulin Measures

Following a 10-h overnight fast, before and after the 10 week-intervention, participants drank a 75 g glucose drink with blood samples taken before, and 30, 60, 90, 120, and 180 min after the drink. Participants’ dietary intake the day before the OGTT at baseline was recorded, and we asked participants to follow a similar dietary pattern at the end of the study. Glucose was measured with an YSI Biochemistry Analyzer (YSI Inc., Yellow Springs, OH, United States) and insulin by ELISA according to the manufacturer’s guidelines (ThermoFisher Scientific); all samples were measured in duplicate. Hemoglobin A1C (HbA1c) values were obtained through a commercial clinical analysis company (LabCorp, United States). Glycated serum protein (GSP), a shorter measure of glycemic control, was assessed by enzymatic assay using manufacturer’s guidelines (Diazyme, Poway, CA, United States). The homeostasis model assessment of insulin resistance (HOMA-IR) was calculated as described previously using the HOMA2-IR online calculator using fasting glucose and insulin levels (27). The Matsuda Index was calculated using glucose and insulin concentrations at Time 0, 30, 60, 90, and 120 min of the OGTT using previously described methods and the online calculator (28, 29). AUCs of the glucose and insulin curves were calculated by the trapezoid method. We included the total AUC and the incremental AUC, which accounts for changes in baseline (Time 0) measurements in response to the exercise intervention (30, 31).

### Statistics

All analyses were conducted using IBM SPSS version 23.0 (Armonk, NY, United States) with all data presented as mean ± standard deviation (SD) unless otherwise stated. The primary outcome of the study was change in aerobic capacity and the secondary outcome was change in neutrophil chemotaxis. We powered the study to detect a 10% change in VO_2__peak_ based on our previous studies, which suggested a minimum of 8 participants (10, 11). Normality was assessed using Kolmogorov-Smirnov analysis; for variables violating normality, natural log transformation was performed. Pairwise comparisons of variables were completed using paired *T*-tests; independent *T*-tests were used to compare the young control group to the older group. Spearman and Pearson correlations (depending on normality) were conducted between percentage changes in fitness, body composition, glucose and insulin, inflammatory markers, and immune functions to assess associations. Results are presented with 95% confidence intervals and effect sizes calculated as Cohen’s *D*. Statistical significance was accepted as *p* ≤ 0.05.

## Results

### HIIT Effects on Health and Function

Diabetes risk factors were improved following training (Table 2). Specifically, fasting glucose (*p* = 0.026, 95% CI 0.03, 0.61, *d* = 0.71) and insulin concentrations (*p* = 0.013, 95% CI 4.4, 28.9, *d* = 0.97) were reduced by 6% and 14%, respectively, resulting in lower insulin resistance (HOMA-IR *p* = 0.007, 95% CI 0.12, 0.56, *d* = 1.09) and increased insulin sensitivity (Matsuda *p* = 0.014, 95% CI −0.62, −0.09, *d* = 0.98). During the 3-h OGTT, glucose (*p* = 0.029, 95% CI 37.2, 306.3, *d* = 1.07) and insulin (*p* = 0.025, 95% CI 18.8, 216.9, *d* = 0.91) total area under the curve (AUC) were reduced by 12 and 9%, respectively. Similarly glucose incremental AUC was reduced (*p* = 0.022, 95% CI 20.7, 197.9, *d* = 0.85), but 2 h glucose (*p* = 0.994, 95% CI −1.7, 1.71, *d* = 0.00) and insulin (*p* = 0.922, 95% CI −15.4, 16.8, *d* = 0.04) were unchanged. Long-term (∼3-months) glycemic control, as measured by HbA1c, showed a trend toward being lower% (*p* = 0.09, 95% CI −0.02, 0.26, *d* = 0.60) and mmol/mol (*p* = 0.061, 95% CI −0.08, 2.88, *d* = 0.68), while short-term (∼6 weeks) glycemic control, as measured by GSP reduced by 25% (*p* = 0.044, 95% CI 9.8, 109.8, *d* = 0.64). Despite the lack of change in body fat percentage, the adipose related hormone leptin reduced by 3% (*p* = 0.04, 95% CI 0.26, 11.36, *d* = 0.75) while adiponectin increased by 24% (*p* < 0.001, 95% CI −1.18, −0.62, *d* = 1.58) resulting in a lower ratio of leptin to adiponectin (*p* < 0.001, 95% CI 8.6, 16.36, *d* = 2.30). NEFA concentrations remained unchanged (*p* = 0.439, 95% CI −0.25, 0.12, *d* = 0.26).

**TABLE 2:** Pre-and Post HIIT values for clinical, metabolic characteristics, and physical functioning.

	**Pre-HIIT**	**Post-HIIT**	***p-*value**	**95% C.I.**	**Effect size (*d*)**
Age (years)	71±5				
Gender (M/F)	4/6				
**Clinical characteristics**					
Weight (kg)	83.3 ± 13.6	82.1 ± 13.4	0.201	−0.77, 3.19	0.43
BMI (kg/m^2^)	29.4 ± 3	29.0 ± 3	0.223	−0.29, 1.09	0.41
Body fat (%)	39.6 ± 8.6	39.1 ± 8.1	0.571	−1.37, 2.33	0.19
Fat free mass (kg)	50.2 ± 12.2	50.2 ± 12.0	0.987	−1.40, 1.40	0.00
**Blood pressure (mmHg)**					
Systolic	129 ± 10	129 ± 12	0.923	−11.9, 10.9	0.03
Diastolic	72 ± 11	67 ± 10	0.125	−1.75, 12.15	0.54
MAP	91 ± 9	87 ± 10	0.298	−3.46, 10.06	0.35
Resting heart rate (bpm)	66 ± 7	64 ± 8	0.406	−2.39, 5.39	0.28
**Metabolic characteristics**					
HbA1c (%)	6 ± 0.2	5.9 ± 0.2	0.09	−0.02, 0.26	0.60
HbA1c (mmol/mol)	42.1 ± 1.7	40.7 ± 2.1	0.061	−0.08, 2.88	0.68
GSP (μmol/L)	196 ± 62	146 ± 33	0.044	9.8, 109.8	0.64
Fasting glucose (mmol/L)	6.04 ± 0.6	5.73 ± 0.6	0.026	0.03, 0.61	0.71
Fasting insulin (pmol/L)	103.3 ± 36.8	92.2 ± 34.6	0.013	4.4, 28.9	0.97
**OGTT**					
Glucose AUC (mmol.min^–1^/L)	1448 ± 239	1277 ± 174	0.029	37.2, 306.3	1.07
Glucose iAUC	586 ± 99	477 ± 152	0.022	20.7, 197.9	0.85
2-h Glucose (mmol/L)	7.72 ± 1.89	7.72 ± 1.65	0.994	−1.70, 1.71	0.00
Insulin AUC (pmol/L)	1416 ± 1421	1298 ± 1438	0.025	18.8, 216.9	0.91
2-h Insulin (pmol/L)	72.9 ± 28.5	72.1 ± 30.4	0.922	−15.4, 16.8	0.04
HOMA-IR	2.3 ± 0.7	1.9 ± 0.7	0.007	0.12, 0.56	1.09
Matsuda index	2.2 ± 0.5	2.5 ± 0.7	0.014	−0.62, −0.09	0.98
Leptin (pg/mL)	238 ± 16	232 ± 19	0.04	0.26, 11.36	0.75
Adiponectin (ng/mL)	3.8 ± 0.2	4.7 ± 0.6	<0.001	−1.18, −0.62	1.58
Leptin: Adiponectin ratio	62 ± 5	50 ± 8	<0.001	8.6, 16.36	2.30
NEFA (mmol/L)	0.4 ± 0.2	0.5 ± 0.1	0.439	−0.25, 0.12	0.26
**Physical function**					
Grip strength (kg)	27 ± 6.8	28.6 ± 5.8	0.112	−3.66, 0.46	0.56
Berg balance score	54.3 ± 5.8	55.0 ± 1.4	0.089	−1.53, 0.13	0.60
TUG (sec)	8.9 ± 1.5	8.4 ± 1.2	0.109	−0.13, 1.07	0.56
400 m walk time (sec)	254 ± 27	238 ± 22	0.004	6.54, 24.86	1.23

*BMI, body mass index; HbA1c, hemoglobin A1c; GSP, glycated serum protein; OGTT, oral glucose tolerance test; AUC, area under curve; iAUC, incremental AUC; HOMA-IR, homeostatic model assessment of insulin resistance; NEFA, non-esterified fatty acids; TUG, timed-up and go. Data are mean ± SD.*

**FIGURE 1:**
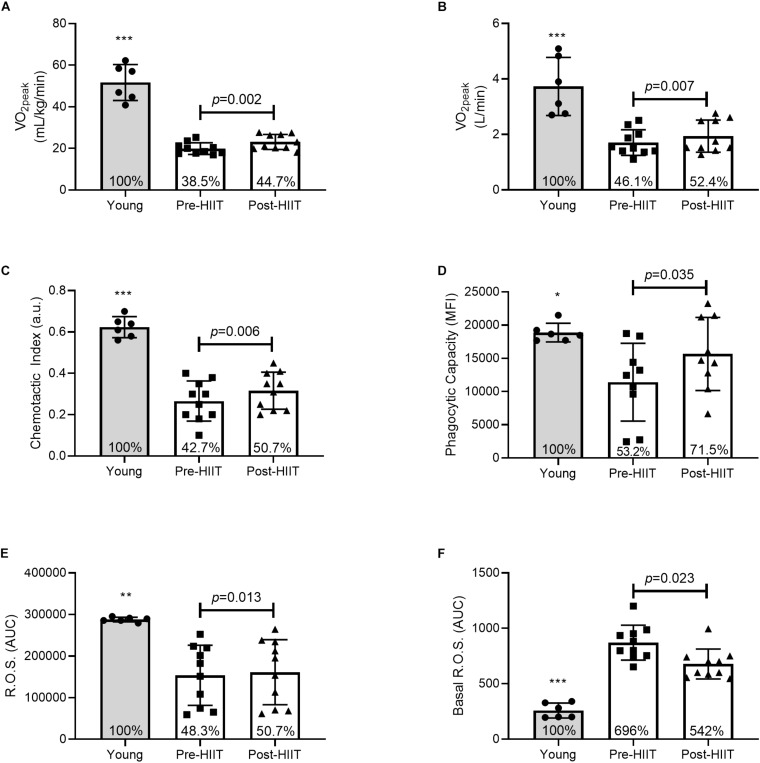
Relative **(A)** and absolute **(B)** cardiorespiratory fitness, neutrophil chemotactic index **(C)**, phagocytic capacity **(D)**, mitogen stimulated reactive oxygen species (ROS) **(E)**, and basal ROS **(F)** differences between young, and older participants before and after 10 weeks of HIIT. The percentages inside each bar represent the percentage of the young healthy control group that was obtained for that variable, with the young group represented as 100%. *p < 0.05, **p < 0.01, and ***p < 0.001 different than older participants at both pre and post HIIT.

Ten weeks of HIIT resulted in a 16 ± 11% (range: 2 – 32%) increase in both relative (Figure 1A: *p* = 0.002, 95% CI −4.8, −1.5, *d* = 1.39) and absolute (Figure 1B: *p* = 0.007, 95% CI −0.38, −0.08, *d* = 1.09) cardiorespiratory fitness. At baseline, older adults had on average 39 and 46% of the relative and absolute fitness of the young controls (both *p* < 0.001), which increased to 44.7 and 52.4%, respectively following training – a cumulative reduction of 6% of the deficit.

As with our previous studies, there were minimal changes in body composition (Table 2). HIIT did not induce significant changes in weight (*p* = 0.201, 95% CI −0.77, 3.19, *d* = 0.43), BMI (*p* = 0.223, 95% CI −0.29, 1.09, *d* = 0.41), body fat% (*p* = 0.571, 95% CI −1.37, 2.33, *d* = 0.19) or lean mass (*p* = 0.987, 95% CI −1.4, 1.4, *d* = 0.00). As such, physical functions typically associated with changes in body composition were unchanged (Table 1). Specifically, grip strength (*p* = 0.112, 95% CI −3.66, 0.46, *d* = 0.56), balance (*p* = 0.089, 95% CI −1.53, 0.13, *d* = 0.60), or the time to stand and walk 6 meters (*p* = 0.109, 95% CI −0.13, 1.07, *d* = 0.56). The time to complete a fast 400 m walk is considered a surrogate for aerobic fitness in older adults (32), and was significantly reduced following training (*p* = 0.004, 95% CI 6.54, 24.86, *d* = 1.23).

### Effects of HIIT on Neutrophil Functions

In response to 10 weeks of HIIT, migration of neutrophils toward the chemokine IL-8 increased by 26%, Figure 1C. Chemotactic accuracy [chemotactic index (*p* = 0.006, 95% CI −0.08, −0.02, *d* = 1.12)] which was driven by improved directional speed [chemotaxis (*p* = 0.013, 95% CI −0.45, −0.07, *d* = 0.97], with no effect on overall speed [chemokinesis (*p* = 0.133, 95% CI −0.53, 0.08, *d* = 0.52)]. Chemotactic index was approximately 43% of the young at baseline (*p* < 0.001), which increased to 51% following training (*p* < 0.001) – a cumulative reduction of 7% of the deficit. Similarly, HIIT increased the amount of FITC-labeled *E. coli* each neutrophil could ingest (Figure 1D: *p* = 0.035, 95% CI −7290, −351, *d* = 0.85). At baseline phagocytosis was 53% of the young (*p* = 0.012), which increased to 71.5% following training (*p* = 0.202) – a cumulative reduction of 18.3% of the deficit. HIIT increased mitogen stimulated ROS production (Figure 1E: *p* = 0.013, 95% CI −12071, −1891, *d* = 0.98), which at baseline was 48% of the young response (*p* = 0.002), increasing to 51% following training (*p* = 0.004) – a cumulative reduction of 2.4% of the deficit. HIIT also reduced basal unstimulated ROS production (Figure 1F: *p* = 0.023, 95% CI 11.25, 114.31, *d* = 0.93), which at baseline was almost 700% higher than young controls (*p* < 0.001), before reducing to 542% of the young following training (*p* < 0.001) – a cumulative improvement of 154% of the deficit. Plasma neutrophil elastase was unchanged following training, data not shown (*p* = 0.120, 95% CI −3.59, 0.50, *d* = 0.85). Functional immune improvements were not associated with changes in cell surface receptor expression of CXCR2, MAC-1, TLR-2, or TLR-4, data not shown (all *p* > 0.05).

**FIGURE 2:**
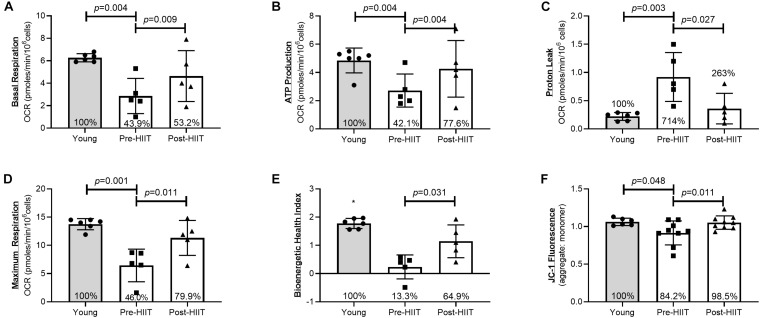
Neutrophil mitochondrial health as assessed by basal respiration **(A)**, ATP production **(B)**, Proton Leak **(C)**, Maximal Mitochondrial Respiration **(D)**, Bioenergetic Health Index **(E)**, and JC-1 ΔΨm fluorescence **(F)** differences between young, and older participants before and after 10 weeks of HIIT. The percentages inside each bar represent the percentage of the young healthy control group that was obtained for that variable, with the young group represented as 100%.*p < 0.06 different than older participants at both pre and post HIIT.

Following 10 weeks of HIIT, neutrophils of older adults increased basal respiration (Figure 2A: *p* = 0.009, 95% CI −31.3, −9.67, *d* = 3.01), which at baseline was 44% of the young response (*p* = 0.004), increasing to 53% following training (*p* = 0.195) – a cumulative reduction of 13.3% of the deficit. HIIT increased ATP production (Figure 2B: *p* = 0.004, 95% CI −26.2, −11.77, *d* = 4.18), which at baseline was 42% of the young response (*p* = 0.004), increasing to 77.6% following training (*p* = 0.234) – a cumulative reduction of 35.5% of the deficit. HIIT reduced proton leak (Figure 2C: *p* = 0.027, 95% CI 1.32, 10.68, *d* = 2.02), which at baseline was 714% of the young response (*p* = 0.003), reducing to 263% following training (*p* = 0.123) – a cumulative reduction of 451% of the deficit. HIIT increased maximum respiration (Figure 2D: *p* = 0.011, 95% CI −73.9, −21.1, *d* = 2.86), which at baseline was 46% of the young response (*p* = 0.001), increasing to 80% following training (*p* = 0.088) – a cumulative reduction of 33% of the deficit. The bioenergetic health index (BHI) is a composite index of mitochondrial functions (33). HIIT increased BHI (Figure 2E: *p* = 0.031, 95% CI −1.92, 0.083, *d* = 1.46), which at baseline was 13% of the young response (*p* < 0.001), increasing to 65% following training (*p* = 0.058) – a cumulative reduction of 51.6% of the deficit. HIIT increased mitochondrial membrane potential, ΔΨm (Figure 2F: *p* = 0.011, 95% CI −0.23, −0.042, *d* = 1.10), which at baseline was 84% of the young response (*p* = 0.048), increasing to 98.5% following training (*p* = 0.817) – a cumulative reduction of 14.3% of the deficit.

### Effects of HIIT on Systemic Inflammation and Immune Cell Numbers

With respect to the systemic inflammatory milieu (Table 3), HIIT did not change concentrations of IL-1β (*p* = 0.763, 95% CI −0.04, 0.03, *d* = 0.00), IL-6 (*p* = 0.404, 95% CI -0.44, 1.00, *d* = 0.30), IL-7 (*p* = 0.728, 95% CI −1.25, 0.90 *d* = 0.13), IL-8 (*p* = 0.109, 95% CI −2.28, 2.74, *d* = 0.56), IL-10 (*p* = 0.786, 95% CI −0.04, 0.05, *d* = 0.00), IL-15 (*p* = 0.774, 95% CI −18, 0.23, *d* = 0.14), TNFα (*p* = 0.279, 95% CI −53, 0.17, *d* = 0.20), or the acute phase protein CRP (*p* = 0.569, 95% CI −1.93, 1.13, *d* = 0.28). Although HIIT did not change circulating numbers of neutrophils (*p* = 0.127, 95% CI −0.08, 0.54, *d* = 0.50), there was a trend for a lower white blood cell count (*p* = 0.059, 95% CI −0.01, 0.53, *d* = 0.53) which was due to a small non-significant reduction in monocytes (*p* = 0.081, 95% CI 0.06, 1.96, *d* = 3.01) and eosinophils (*p* = 0.081, 95% CI 0.06, 1.96, *d* = 0.55).

**TABLE 3:** Pre-and Post HIIT fasting blood values for systemic cytokine concentrations and white blood cell counts.

	**Pre-HIIT**	**Post-HIIT**	***p-*value**	**95% C.I.**	**Effect size (*d*)**
**Inflammation (pg/mL)**					
IL-1β	0.1 ± 0.1	0.1 ± 0.1	0.763	−0.04, 0.03	0.00
IL-6	1.2 ± 1.0	0.9 ± 0.6	0.404	−0.44, 1.00	0.30
IL-7	2.9 ± 1.7	3.1 ± 1.6	0.728	−1.25, 0.90	0.13
IL-8	8.8 ± 4.2	9.8 ± 3.9	0.109	−2.28, 2.74	0.56
IL-10	0.3 ± 0.1	0.3 ± 0.1	0.786	−0.04, 0.05	0.00
IL-15	3.1 ± 0.9	3.2 ± 1.2	0.774	−0.18, 0.23	0.14
TNFa	2.9 ± 0.9	3.0 ± 1.1	0.279	−0.53, 0.17	0.20
CRP (mg/L)	1.7 ± 2.4	2.1 ± 1.7	0.569	−1.93, 1.13	0.28
**WBC (×l0^9^/L)**					
Total	5.4 ± 1.5	5.2 ± 1.4	0.059	−0.01, 0.53	0.53
Neutrophils	2.9 ± 1.1	2.7 ± 1.2	0.127	−0.08, 0.54	0.50
Lymphocytes	1.8 ± 0.6	1.9 ± 0.6	0.849	−0.38, 0.32	0.20
Monocytes	0.5 ± 0.1	0.4 ± 0.1	0.081	0.06, 1.96	3.01
Eosinophils	0.21 ± 0.2	0.18 ± 0.2	0.081	0.06, 1.96	0.55

*IL, interleukin; TNFα, tumor necrosis factor α; CRP, C – reactive protein; WBC, white blood cell. Data are mean ± SD.*

### Associations Between Changes in Fitness, Glucose Control, and Immune Function

**FIGURE 3:**
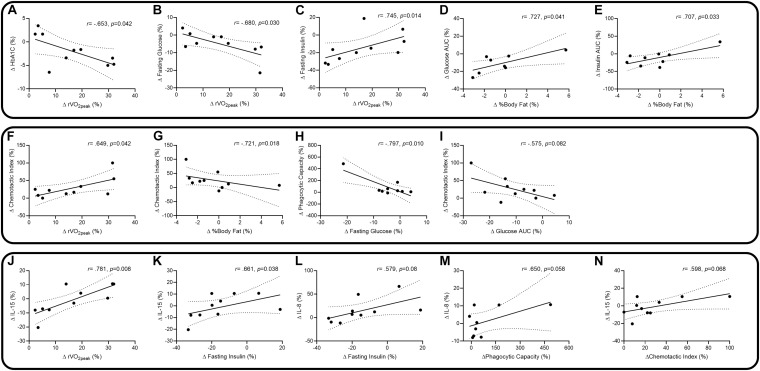
Spearman and Pearson correlations, with 95% confidence intervals, between percentage changes in relative peak VO_2_ (rVO_2peak_) and HbA1c **(A)**, rVO_2peak_ and fasting glucose **(B)**, rVO_2peak_ and fasting insulin **(C)**, body fat% and glucose total AUC **(D)**, body fat% and insulin total AUC **(E)**, rVO_2peak_ and chemotaxis **(F)**, body fat% and chemotaxis **(G)**, fasting glucose and phagocytosis **(H)**, glucose total AUC and chemotaxis **(I)**, rVO_2peak_ and IL-15 **(J)**, fasting insulin and IL-15 **(K)**, fasting insulin and IL-8 **(L)**, phagocytosis and IL-8 **(M)**, and chemotaxis and IL-15 **(N)**.

Correlations between changes in outcome measures are presented in Figure 3. Changes in physiology and diabetes factors suggest there were negative correlations between percentage change in relative VO_2__peak_ (rVO_2__peak_) and percentage changes in HbA1c (Figure 3A: *r* = −0.653, *p* = 0.042) and fasting glucose (Figure 3B: *r* = −0.680, *p* = 0.030). There were positive correlations with rVO_2__peak_ and changes in fasting insulin (Figure 3C: *r* = 0.745, *p* = 0.014), body fat percentage and glucose total AUC (Figure 3D: *r* = 0.727, *p* = 0.041) and body fat and insulin total AUC (Figure 3E: *r* = 0.707, *p* = 0.033). Changes in physiology and diabetes factors associated with neutrophil functions suggest there was a positive correlation between change in rVO_2__peak_ and neutrophil chemotaxis (Figure 3F: *r* = 0.649, *p* = 0.042), and negative correlations with changes in body fat and chemotaxis (Figure 3G: *r* = −0.721, *p* = 0.018), changes in fasting glucose and phagocytosis (Figure 3H: *r* = −0.797, *p* = 0.010), and a non-significant trend in changes of glucose total AUC and chemotaxis (Figure 3I: *r* = −0.575, *p* = 0.082). Changes in physiology, diabetes factors, and neutrophil functions associated with changes in IL-8 and IL-15 suggest positive correlations with changes in rVO_2__peak_ and IL-15 (Figure 3J: *r* = 0.781, *p* = 0.008), fasting insulin and IL-15 (Figure 3K: *r* = 0.661, *p* = 0.038), and non-significant positive trends between fasting insulin and IL-8 (Figure 3L: *r* = 0.579, *p* = 0.08), phagocytosis and IL-8 (Figure 3M: *r* = 0.650, *p* = 0.058) and chemotaxis and IL-15 (Figure 3N: *r* = 0.598, *p* = 0.068). Each of the correlations for rVO_2__peak_ were similarly observed for changes in absolute VO_2__peak_ (data not shown).

## Discussion

In older (aged 65–80 years) adults at risk for type 2 diabetes, 10 weeks of high-intensity interval walking at levels exceeding exercise guidelines (12), was associated with reducing risk factors associated with the development of type 2 diabetes, increased aerobic fitness and physical function, and improved neutrophil primary and bioenergetic functions. Compared to young healthy adults, cardiorespiratory fitness, glucose and insulin, and neutrophil improvements suggested a change, or rejuvenation, toward functions of younger adults. Correlative assessment of changes in variables suggest an intricate interplay where cardiorespiratory fitness increases are associated with beneficial changes in glucose homeostasis, neutrophil chemotaxis and IL-15 concentrations. Further, changes in body fat percentages were also associated with changes in glucose homeostasis and neutrophil chemotaxis. Changes in glucose homeostasis were associated with beneficial changes in neutrophil phagocytic capacity, chemotaxis, and the cytokines IL-8 and IL-15.

### HIIT and Neutrophil Function in Prediabetes

Type 2 diabetes and its precursor, prediabetes, are characterized by impaired insulin sensitivity and increasing circulating blood glucose concentrations. The similarly increased glucose-mediated advanced glycation end products are associated with elevated unstimulated neutrophil ROS production but also with lower stimulated ROS production (34). These data suggest that neutrophils are adding to systemic inflammation by chronic ROS production, which leads to tissue damage. Further, because they have impaired ROS during pathogenic activation, risk of infection is greater and time to resolve infections is longer. In those at risk for diabetes, neutrophil chemotaxis is also impaired (35), which was confirmed here, contributing to inflammation as reduced migratory accuracy leads to increased protease-mediated tissue damage during migration (22).

Few studies have assessed neutrophil functions in response to exercise, fewer in response to more than 8 weeks of HIIT, even fewer in prediabetes, and none have combined all three in the same study. Robinson and colleagues suggest that along with changes in monocytes, neutrophil expression of TLR-4 is reduced following only 10 days of HIIT in a slightly younger cohort of individuals with prediabetes than in our study (13). Although it is unclear whether reduced TLR-4 on neutrophils was a positive outcome, the reductions for monocyte TLR-4 are typically accepted as being anti-inflammatory in nature. We observed similar reductions of monocyte TLR-4 but not neutrophil TLR-4 in our recent HIIT study in rheumatoid arthritis (RA) patients (11). Similar to observations in healthy adults (10), in those with prediabetes, several key neutrophil functions were improved following 10 weeks of HIIT.

Although our sample size was small, the correlations with glucose homeostasis and improved phagocytosis and chemotaxis suggest an interplay with glucose and neutrophil functions. Increasing glucose concentrations are associated with impaired neutrophil chemotaxis and phagocytosis (36, 37). In hyperglycemic patients, neutrophil functions can be restored by lowering glucose with insulin treatment (38, 39). As such, insulin sensitivity is the likely regulator of neutrophil functions in prediabetic older adults (40). Our correlative analyses suggest relationships with changes in both cardiorespiratory fitness and body fat with changes in glucose homeostasis and insulin sensitivity that likely confer improvements in neutrophil functions. The positive correlation with changes in cardiorespiratory fitness and chemotaxis suggest that at least part of the effect was driven by changes in fitness induced by HIIT. However, changes in body fat percentage were also associated with beneficial changes in chemotaxis. Whether fat changes were a direct response of exercise or whether participants engaged in dietary modification is not known. We advised participants to not change their diet, but did not track food logs during the intervention. Regardless, the intervention induced significant changes in fitness, and small non-significant changes in body composition which was related to improved glucose control and insulin sensitivity, and neutrophil functions in a high infection risk population. Although we cannot ascertain whether this represents a reduced risk of opportunistic microbial infections, this is the likely outcome of such improved neutrophil bactericidal function.

### HIIT and Neutrophil Bioenergetics in Prediabetes

The underlying mechanisms of exercise-induced neutrophil improvements are not well defined, and likely multifactorial. Neutrophils are terminally differentiated and have low transcriptional activities compared to other immune cells (41). Immune cells are reliant on metabolic regulation for optimal functions during activation (42). Changes in activation, such as migration to sites of inflammation require significant metabolic demands (42, 43). Compared to T-cells, neutrophil metabolic pathways and metabolite availability are not as clearly understood. The common dogma holds that neutrophils from young healthy individuals rely almost exclusively on glycolysis for energy production, with little to no need for mitochondrial energy production (3, 44, 45). Glycolysis is essential for optimal function at sites of inflammation, which are typically hypoxic (46). However, suggesting impaired glycolysis and more reliance on mitochondrial produced ATP, both aging and diabetes are associated with reduced ATP and lactate production (45, 47). Chemical uncoupling of mitochondrial oxidative phosphorylation with FCCP results in impaired neutrophil chemotaxis, but not activation of respiratory burst or phagocytosis (48). Therefore, neutrophil metabolism may be regulated according to specific function (e.g., phagocytosis and chemotaxis), while perturbations to health (e.g., exercise, diabetes and aging) may alter neutrophil metabolic regulation (44, 45, 48).

Syu and colleagues describe young healthy men with neutrophil mitochondrial changes following a single bout of exercise and 4 months of chronic exercise (49, 50). Exercise training improved neutrophil phagocytosis and chemotaxis in association with improved mitochondrial membrane potential (ΔΨm). Mooren and colleagues suggest acute exercise does not alter ΔΨm, but the acute increase in the neutrophil growth factor G-CSF was associated with delaying apoptosis and improving calcium flux mediated functions (51). Single acute bouts of exercise mobilizes neutrophils into the peripheral blood before they leave the blood upon completion of exercise (52). These cells are characterized by differences in gene expression and heightened effector functions (53, 54). After only 1 h of cycling followed by 1 h of running, young healthy men’s neutrophils share similar mitochondrial gene networks as skeletal muscle (54). Therefore, exercise may serve as a stimulus influencing mitochondrial function in neutrophils; each bout of exercise may modify neutrophil mitochondria resulting in enhanced bioenergetics following an exercise training program.

For the first time we have observed, compared to healthy young adults, prediabetes in older adults was associated with impaired neutrophil mitochondrial functions. Similar to changes in cardiorespiratory fitness and primary neutrophil functions, 10 weeks of HIIT improved mitochondrial functions toward those of younger adults. It is unclear whether our results are indicative of enhanced mitochondrial biogenesis or comprehensive qualitative improvements in individual mitochondria. Neutrophils possess a complex mitochondrial network extending through the cytoplasm. Similar to Syu and colleagues, we observed an improvement in the ΔΨm, which was associated with enhanced chemotaxis (49, 50). ΔΨm is critical to ATP production by ATP synthase and a measure of mitochondria viability (55). ΔΨm improvements were also associated with increases in ATP production and lower proton leak. Future research should aim to understand the relationships with exercise training and neutrophil bioenergetics in order to determine the precise mechanisms by which exercise provides enhanced functions.

### HIIT and Inflammation in Prediabetes

Although the underlying pathogenesis of prediabetes and type 2 diabetes is considered to include tissue exposure to chronic inflammation, we did not observe any changes for typical inflammatory measures following HIIT. Considering the majority of exercise studies that robustly alter plasma cytokines and acute phase protein levels are associated with weight loss, this did not come as a surprise. Indeed, in our previous studies in patients with RA and sedentary healthy adults where we observed minimal weight loss following HIIT, we did not observe changes in cytokine concentrations (10, 11). Our correlative analyses suggested relationships with changes in cardiorespiratory fitness, insulin, and the cytokines IL-8 and IL-15. Both of these cytokines play prominent roles in the functions of neutrophils. IL-15 is expressed constitutively in many cells and tissues including skeletal muscle and heart, monocytes and macrophages (56), while IL-8 is secreted primarily by neutrophils and monocyte/macrophages. Unlike IL-8, which neutrophils will migrate toward and produce a respiratory burst response (9, 25), IL-15 enhances functions by optimizing cytoskeletal rearrangement and cellular morphology (57). IL-15 co-activates the neutrophil which in turn promotes enhanced phagocytosis, and *de novo* protein synthesis including IL-8 (58, 59). We recently showed that IL-15 concentrations are higher in physically active healthy older adults, and was associated with improved CD4+ T-cell phenotypes (60). Further, IL-15 is secreted by muscle during exercise, and at higher concentrations promotes metabolic adaptations including improved insulin sensitivity in tissues (61, 62). In our previous cross-sectional analyses, we did not assess IL-15, but suggested a relationship with fasting insulin and neutrophil chemotaxis (9). Although we did not observe changes with insulin and chemotaxis in the current study, the relationships with change in fitness and IL-15, insulin and both IL-15 and IL-8, IL-15 and chemotaxis, and IL-8 and phagocytosis here suggest an intricate relationship with exercise, metabolism and inflammation which may enhance neutrophil functions.

We and others have observed that HIIT alters innate immune cell phenotype, reducing expression of typically pro-inflammatory receptors on monocytes (11, 13). Monocytes migrate to adipose tissue, differentiate into M1 macrophages, and are key features of adipose tissue inflammation. We observed a reduction in leptin and a coincident increase in adiponectin following HIIT. These two hormones are produced predominantly by adipose cells, and reflect both size and function of the adipose tissue. Therefore, although we did not observe significant reductions in fat mass or inflammation, it is likely HIIT altered adipose tissue function, perhaps preparing the tissue for reductions in mass and inflammatory potential. Future studies aimed at assessing exercise effects on inflammation should assess either tissue (e.g., muscle, adipose, and bone marrow), cells of interest, or robust measures of inflammation such as GlycA (7), rather than only systemic plasma cytokines.

### Limitations and Future Directions

Our study is limited by the lack of an age-matched healthy control group. We are unable to specify that improvements in disease risk and immune function were unique to prediabetes. In fact it is likely the magnitude of improvements are not unique to prediabetes. We previously reported immune improvements in both sedentary healthy adults (20–60 year) and older adults with rheumatoid arthritis (52–78 year) using a similar exercise prescription. Therefore it is likely that this specific exercise prescription is capable of bypassing pathological (age, autoimmune, metabolic) related innate immune dysfunction. Future randomized control studies that aim to understand the molecular mechanisms and the direct relationships with disease pathology and innate immune enhancements are needed.

Another limitation of our study is that we did not randomize prediabetic participants to an exercise or a control group, thus we are unable to specify that improvements in disease risk and immune function resulted directly from the exercise. However, the landmark DPP study (63) indicated that when prediabetic adults do not undertake lifestyle modifications or 1700 mg/day metformin they have significantly higher risk of developing diabetes through worsening glucose control. Similarly, Sapey and colleagues (22) highlighted that neutrophil functions become progressively worse with age and do not appear to spontaneously improve. Therefore we are confident that the improvements in glucose control and immune function are a result of our exercise prescription. Future studies should aim to extend the period of HIIT training to 6-months and comprehensively assess diabetes and cardiometabolic risk factors. Further, it would be extremely useful to understand the legacy effects of this type training, i.e., how long does the effect last after the training is completed?

Although this study was a pilot, it is unclear whether such an intervention would be feasible and provide similar results in an older group with greater diabetes disease. Further, this was a supervised, structured exercise intervention; it is not clear whether these results generalize to non-supervised, community-based exercise. Future studies should address a broader population and determine whether this program can be feasibly transferred to a community- or home-based setting.

We are confident that a significant portion of the effects were due to the intervention and that participants were not engaging in extra exercise or diet outside our study design. However, we cannot be certain this did not occur. We analyzed validated questionnaires—the Incidental and Planned Activity Questionnaire and the Stanford Brief Activity Survey—and quantifiable accelerometer (Actigraph) data; we found no differences between time points (data not shown). We did not assess dietary changes during the study, and only counseled participants to not change their diet. In light of the lack of change in body composition, we are confident that participants did not change their day-to-day dietary patterns. That being said, we did not control participants dietary intake during the intervention – instead we recommended that they did not change their diet or begin any caloric restriction diets. Acute and chronic changes to glycemic load can change immune cell functions. Specifically, increasing glucose concentrations are associated with impaired neutrophil chemotaxis and phagocytosis (36, 37). Therefore, future exercise studies assessing immune cell functions, should also aim to assess dietary intake during the intervention.

## Conclusion

We observed, for the first time to our knowledge, that 10 weeks of HIIT in older adults with prediabetes improved glucose homeostasis and insulin sensitivity; and these beneficial responses were accompanied by improved neutrophil functions and bioenergetics. Our data suggest that HIIT walking should be an efficient, tolerable, and highly effective intervention to attenuate disease risk and improve overall health, by improving innate immune functions in older adults with prediabetes. However, this should be studied in longer studies with more participants.

## Data Availability Statement

The datasets generated during the present study are not publicly available, owing to the risk of disclosure or deduction of private individual information, but they are available from the corresponding author on reasonable request.

## Ethics Statement

The studies involving human participants were reviewed and approved by the Duke University Medical Center Institutional Review Board. The patients/participants provided their written informed consent to participate in this study.

## Author Contributions

DB, JL, WK, and CS conceived and designed the study and experimental approach. DB performed the immunological experiments and wrote the manuscript. GS analyzed results in a blinded fashion. DB and JM conducted the microscopy. DB, LW, CS, and AH performed the physiological and functional testing and exercise training of participants. CS and AH completed the glucose assessments. DM and TK assisted with Seahorse analyses. JH, VK, and HW completed the cytokine analysis. MM completed the plasma fatty acid analysis. KH assisted in statistical analyses. All authors contributed to critical revisions and approval of the final manuscript.

## Conflict of Interest

The authors declare that the research was conducted in the absence of any commercial or financial relationships that could be construed as a potential conflict of interest.
